# Functional Polymers for Controlled Drug Release

**DOI:** 10.3390/pharmaceutics12020135

**Published:** 2020-02-05

**Authors:** Umile Gianfranco Spizzirri

**Affiliations:** Department of Pharmacy, Health and Nutritional Sciences, University of Calabria, 87036 Rende (CS), Italy; g.spizzirri@unical.it; Tel.: +39-0984-493-298

In the last decade, the pharmaceutical application of hydrophilic materials has emerged as one of the most significant trends in the biomedical and pharmaceutical areas [1]. This Special Issue serves to highlight and capture the contemporary progress recorded in this field.

In this Special Issue, two articles were devoted to exploiting the employment of polyvinyl alcohol in the developing of useful drug delivery tools. Polyvinyl alcohol is one of the most popular water-soluble, non-carcinogenic, biocompatible, biodegradable synthetic polymers, and is largely employed to prepare hydrogels useful as artificial organs, drug delivery devices, and wound dressings [2]. Specifically, Han et al. explored the release properties of lutein-loaded polyvinyl alcohol combined with sodium alginate nanofibers, prepared by electrospinning [3]. The release profiles were analyzed by mathematical models, highlighting that the employment of the electrospinning in the encapsulation of the carotenoid molecule is an effective method to achieve the sustained lutein release. Additionally, Avila-Salas et al. crosslinked polyvinyl alcohol with different dicarboxylic acids to synthesize dressing hydrogels [4]. These formulations were suggested as multi-target therapies in wound healing, as a consequence of the sustained release of simultaneous bioactive compounds, such as dexpanthenol, allantoin, caffeic acid, and resveratrol.

The transport and sustained release of bioactive polyphenols, usually extracted from plants or food, was also dealt with by Guzman-Oyarzo et al. [5]. In order to avoid degradation reactions of these bioactive molecules, the authors proposed a synthetic strategy involving a flexible and soft β-ciclodextrin polymer within the highly porous inorganic matrix of nanoporous silicon [6] as a substrate. This device was tested as carrier for the controlled release of caffeic acid and pinocembrin, two of the main components of a *Chilean propolis* with anti-atherogenic and anti-angiogenic activities. 

The fast release of selected antioxidants and skin-lightening agents by suitable micellar systems, was exploited by Odrobinska et al. for applications in cosmetology as components of masks, creams, and wraps [7]. The authors proposed the synthesis of an innovative material obtained by “click” chemistry reaction of azide-functionalized polyethylene glycol onto multifunctional polymethacrylates containing alkyne units, and using bromoester-modified retinol as the initiator. The tendency of the designed amphiphilic graft copolymers to form micelles allowed them to record a high effective encapsulation of arbutin or vitamin C and in vitro experiments highlighted the maximum release in few minutes. 

Intelligent polymeric devices able to undergo morphological modifications in response to an internal or external stimulus, such as pH, redox balance, temperature, magnetic field, and light have been actively pursued [8,9,10]. In particular, in this Special Issue, Partheniadis et al. [11] synthesized pharmaceutical pellets [12] of different sizes, using an extrusion/spheronization technique, and medium viscosity chitosan for the pH-dependent delivery of piroxicam. The authors suggested that a remarkable reduction in pellet size influenced the release rate, avoiding the need to employ hydrophilic excipients such as lactose [13].

In another paper of this Special Issue, Wang et al. explored a novel strategy to drive the reversible adsorption of peptide-based therapeutics using commercially available contact lenses [14]. To accomplish this, thermo-sensitive elastin-like polypeptides, alone or tagged with a candidate ocular therapeutic, were characterized. This research suggests that elastin-like polypeptides may be useful to control loading or release from suitable formulations, with the aim to deliver appropriate biologically active peptides to the ocular surface via contact lenses.

Finally, this Special Issue was completed by three reviews exploiting the employment of particular materials and/or analyzing specific route of drug administration. In particular, Neugebauer et al. investigated the synthesis of ionic polymethacrylate-based delivery systems, including conjugates and self-assemblies [15]. The influence of the hydrophilic/hydrophobic content on physicochemical and delivery properties of the polymer carriers were exploited, by analysing how the topology and architecture of the macromolecular devices regulate the physical entrapment or chemical attachment of the specific drugs. 

Furthermore, Tomeh et al. analyzed the use of silk fibroin to prepare versatile drug delivery devices [16]. Mild aqueous possessing conditions, high biocompatibility and biodegradability, and the ability to enhance the stability of the loaded active pharmaceutical ingredients, justify the increased use of these natural polymers in the pharmaceutical and biomedical fields [17].

Finally, Cirillo et al. proposed a review focused on the recent advances in the development of highly engineered injectable delivery vehicle systems, suitable for combined chemo- and radio-therapy, as well as thermal and photo-thermal ablation, with the aim of finding effective solutions to overcome the current obstacles of conventional therapeutic protocols [18].
